# Serum 25-hydroxyvitamin D concentrations in 16-year-old Icelandic adolescent and its association with bone mineral density

**DOI:** 10.1017/S1368980019004142

**Published:** 2020-06

**Authors:** SL Gudmundsdottir, H Hrafnkelsson, EL Sigurdsson, E Johannsson

**Affiliations:** 1 Center for Sport and Health Science, School of Education, University of Iceland, Reykjavik, Iceland; 2 Development Centre for Primary Health Care in Iceland, Hafnarfjordur, Iceland; 3 Department of Family Medicine, University of Iceland, Reykjavik, Iceland; 4 Department of Sport and Physical Activity, Western Norway University of Applied Sciences, Bergen, Norway

**Keywords:** 25-hydroxyvitamin D, Bone mineral density, Adolescent, Cross sectional, Vitamin D

## Abstract

**Objective:**

The aim of the study was to assess the potential association between serum 25-hydroxyvitamin D (25(OH)D) and whole-body bone mineral density (BMD) among 16-year-old adolescents and to study the prevalence of 25(OH)D insufficiency, defined as concentration under 50 nmol/l.

**Design:**

A cross-sectional study.

**Setting:**

Reykjavik, Iceland, latitude 64°08′N. Measurements took place in the Icelandic Heart Association's research lab during April–June 2015.

**Participants:**

In total, 411 students in Reykjavik, Iceland, were invited to participate, 315 accepted the invitation (76·6 %) and 289 had valid data (mainly Caucasian).

**Results:**

25(OH)D < 50 nmol/l was observed in 70 % of girls and 66·7 % of boys. 25(OH)D ≥ 50 nmol/l was significantly associated with higher whole-body BMD after adjusting for the influence of sex, height, fat mass and lean mass. A linear relationship between 25(OH)D and whole-body BMD was significant for 25(OH)D < 50 nmol/l (*n* 199, *P* < 0·05) but NS for 25(OH)D ≥ 50 nmol/l (*n* 86, *P* = 0·48).

**Conclusions:**

Our results are in line with some but not all previous studies on the relationship between BMD and 25(OH)D in adolescents. The observed difference in BMD between those with above *v.* below a 25(OH)D concentration of 50 nmol/l was of about a fifth of one SD, which may have a clinical relevance as one SD decrease in volumetric BMD has been associated with a 89 % increase in 2 years risk of fracture. Icelandic adolescents should be encouraged to increase their vitamin D intake as it is possible that their current intake is insufficient to achieve optimal peak bone mass.

Vitamin D affects bone remodelling and is necessary for healthy bone tissue. Recommended serum concentrations of 25-hydroxyvitamin D (25(OH)D) for adults and healthy children to maintain good bone health and decrease risk of fractures in old age are 40–50 nmol/l^(1,2)^. Rickets was eradicated in Europe with discoveries of the positive effects of sunlight and vitamin D in the 19th and 20th centuries^(3)^. Low 25(OH)D concentrations are of concern in many countries^(4)^ as research indicates that worldwide, less than half of the population has sufficient 25(OH)D, at least during winter^(5)^. In studies of Icelandic children born in 1999, our research group reported that at the age of 7 and 9, 65 % and 66 %, respectively, had 25(OH)D concentration under 50 nmol/l^(6,7)^ but in another cohort of 6-year-old children born in 2005, this proportion was 36 %^(8)^.

About one-third of peak bone mass is formed during the years of sexual maturation and peak growth velocity^(9,10)^. Low peak bone mass is thought to be associated with risk of osteoporosis and fractures later in life^(11)^. The association between 25(OH)D and bone mineral density (BMD) among adolescents is uncertain, and study results are inconsistent. A recent Australian study suggests an association between the trajectories of 25(OH)D from childhood to early adulthood and bone mass at age 20 among males but not females^(12)^. On the other hand, a US study found lower BMD among teenage girls with low 25(OH)D, but the association was not observed among boys^(13)^. A Finnish study that included girls only found that 25(OH)D deficiency was associated with significantly lower BMD measured in the distal radius and tibia shaft^(14)^. Conversely, a previous Icelandic study did not find an association between 25(OH)D and BMD among 16–20-year-old females^(15)^.

Considering the northern latitude of Reykjavik, Iceland (64°08′N), the 25(OH)D concentration is likely to be low in the inhabitants, at least during winter. Whether this has any relationship with low BMD, and in particular the high incidence of fractures in the Nordic countries has not been excluded. Our previous study indicates that although more than 60 % of the children in the 1999 birth year cohort had inadequate 25(OH)D concentration at ages 7 and 9, no association was observed with BMD or bone accrual during a 2-year follow-up^(16)^. In 2015, a health study of the 1999 birth year cohort was performed, including many of the participants from our former studies, as well as additional subjects. It was therefore of interest to investigate the association between 25(OH)D and BMD following sexual maturation and peak growth velocity. Serum 25(OH)D < 50 nmol/l is globally accepted as insufficient vitamin D status^(17)^. Hence, the main aim of the current study was to assess the potential cross-sectional association between serum 25(OH)D and whole-body BMD among 16-year-old adolescents and to study 25(OH)D concentration in this same cohort, with the aim of study the prevalence of 25(OH)D insufficiency, defined as <50 nmol/l, in line with other Nordic and European countries^(18,19)^.

## Methods

This is a cross-sectional study based on data collected during April–June 2015. Students in 10th grade in the same six schools as were included in a previous study^(16)^ were invited to participate. Some of the previous participants had moved and new students enrolled since the last data collection at the age of 9. Thus, this study includes many of the participants from the former study with additional students invited to the current one. A total of 411 students were invited by letter to participate and 315 accepted the invitation (76·6 % participation rate). Measurements took place in the Icelandic Heart Association's research lab in Kopavogur. This study was conducted according to the guidelines laid down in the Declaration of Helsinki, and all procedures involving research study participants were approved by The National Bioethics Committee. Written informed consent was obtained from all adolescents and their guardians before participation.

Body weight (kg) was measured to the nearest 0·1 kg on a calibrated scale (Seca model 813; Seca Ltd.), with participants wearing light clothing and standing height (m) to the nearest mm with a stadiometer (Seca model 217; Seca Ltd.). BMI was determined by dividing weight by height squared (kg/m^2^). Participants were classified as normal, overweight or obese as suggested by Cole *et al.* ^(20)^.

Fasting venous blood samples were collected with a Vacutainer^®^ System (Becton Dickinson). Samples were centrifuged within 30 min and stored at −80°C until analysis when it was thawed at room temperature (only thawed once). The measure of the quantitative determination of total 25(OH)D (nmol/l) was made on Cobas e 411 (Roche) using the vitamin D total assay from Roche, a competitive electrochemiluminescence-binding assay. Calibration was done with the vitamin D total Calset (standard). The inter-assay CV was <6·34 % when calculated data are from measurements using a frozen serum pool as the control sample and <12·80 % when calculated data are from measurements using Roche quality controls.

Intact parathyroid hormone (PTH) (ng/l) in serum was measured using the PTH-intact assay from Roche, a sandwich electrochemiluminescence immunoassay ‘ECLIA’ on Cobas e 411 (Roche, Switzerland). The inter-assay CV was 6·0 % using a frozen serum pool and <4·8 % using quality control samples from Roche.

Whole-body bone mineral density and bone mineral content (BMC), fat and lean tissue composition was measured by dual-energy X-ray absorptiometry (DXA) with Lunar bone densitometer (Lunar iDXA, General Electric Healthcare). Due to financial constraints, regional sites were not assessed. All DXA-scans were run by a single certified radiologist. Daily quality assessment included a scan of the standard calibration image quality assurance (IQA) phantom (Lunar iDXA) that includes different densities (Lunar iDXA). The daily phantom scans demonstrated very stable scanner over the course of the study period with an average CV of 0·19 % for BMD.

Descriptive summaries are presented as means and SDs for continuous variables. Differences between the sexes were evaluated by unpaired *t*-tests. The association between serum 25(OH)D and PTH was assessed with Pearson's correlation coefficient. Linear regression model was performed with whole-body BMD as the dependent factor and 25(OH)D as independent factor. Based primarily on a priori considerations, sex, height, fat mass and lean body mass were considered as covariates^(21)^. Collinearity between lean and fat mass was assessed and found non-significant. There was however a significant correlation between 25(OH)D and PTH, and hence, PTH was not included in the model. Thus, we could evaluate the association between 25(OH)D and whole-body BMD independent of potential confounding factors. Regression analyses were performed for the total group to preserve statistical power instead of for each sex specifically. In the linear regression, subjects were classified according to their 25(OH)D concentration, <50 *v.* **≥**50 nmol/l in accordance with guidelines from i.a. the Icelandic Directorate of Health^(1)^ and to enable comparison with previous studies of the Icelandic paediatric population^(6,7,16)^. An additional analysis treating 25(OH)D as a continuous variable was run to confirm the former. To investigate if a 25(OH)D concentration of **<**50 nmol/l is indeed a meaningful threshold value, we stratified the sample by this value and investigated the linear relationship between 25(OH)D and whole-body BMD adjusting for the aforementioned parameters. This was repeated for 25(OH)D < 37·5 nmol/l. Significant differences or relations were accepted at *α* < 0·05. Statistical analyses were performed using the statistical software package IBM SPSS statistics, version 24 (IBM Corporation).

## Results

Out of the 315 adolescents participating in the study, 307 had DXA measurements and 293 had valid 25(OH)D measures, 289 had valid measures on all parameters included in the analyses. Subject characteristics are shown in Table 1. 25(OH)D < 50 nmol/l was observed in 70 % of girls and 66·7 % of boys.

**Table 1:** Characteristics of study participants

	Females (*n* 183)		Males (*n* 132)		Difference between the sexes
	Mean	sd	Mean	sd	*P* value
Age	15·9	0·3	15·8	0·3	0·09
Height (cm)	166·8	5·7	178·2	5·7	<0·001
Weight (kg)	61·7	10·1	69·0	11·3	<0·001
BMI (kg/m^2^)	22·2	3·2	21·7	3·3	0·240
Normal weight (%)	79·1		81·3		<0·05
Overweight (%)	17·6		14·0		
Obesity (%)	3·3		4·7		
Fat mass (kg)	19·0	6·7	13·4	7·8	<0·001
Fat (%)	31·3	6·2	19·3	7·7	<0·001
Lean body mass (kg)	40·4	4·8	53·0	6·3	<0·001
Bone mass (kg)	2·3	0·3	2·8	0·4	<0·001
Bone mineral density (g/cm^2^)	1·101	0·089	1·138	0·107	0·001
PTH (ng/l)	39·4	13·3	42·2	14·1	0·08
25(OH)D (nmol/l)	43·1	23·0	42·0	22·7	0·67
25(OH)D < 50 nmol/l *n* (%)	70		66·7		0·54

The adjusted associations observed between the lower *v.* higher category of 25(OH)D and whole-body BMD are shown in Table 2. 25(OH)D equal to or above 50 nmol/l was significantly associated with higher whole-body BMD after adjusting for the influence of sex, height, fat mass and lean mass. Of the covariates included in the model, all but fat mass were significantly associated with whole-body BMD. Comparable results were found when 25(OH)D was treated as a continuous variable, a positive association was observed with increasing 25(OH)D values and higher whole-body BMD (*P* = 0·01, data not shown). When stratifying the sample by 25(OH)D status, we found a significant adjusted relationship between 25(OH)D < 50 nmol/l (*n* 199, *P* < 0·05) and whole-body BMD, while the association was NS for those with 25(OH)D > 50 nmol/l (*n* 86, *P* = 0·48). The association was similar when the threshold concentration was set to <37·5 nmol/l (*n* 143, *P* < 0·05).

**Table 2:** Linear regression relationship between 25(OH)D nmol/l and whole-body bone mineral density (g/cm^2^)

	*B*	*β*	se	*P*
25(OH)D **≥ **50 nmol/l	0·023	0·109	0·009	0·009
Sex (male)	0·124	0·015	0·623	<0·001
Height (cm)	−0·002	−0·174	0·001	0·015
Fat mass (kg)	−0·001	−0·050	0·001	0·293
Lean mass (kg)	0·014	1·197	0·001	<0·001
*R* ^2 ^= 0·534				

*B*, unstandardised regression coefficient; *β*, standardised coefficient.

There was also a significant association between higher BMC and increasing 25(OH)D concentration when adjusting for sex, height, fat mass, lean body mass and bone area (*P* = 0·03). No significant association was found between 25(OH)D and bone area. There was a significant negative correlation between 25(OH)D and PTH (*r* = −0·39, *P* < 0·01).

## Discussion

In the current study, we aimed to study the concentration of serum 25(OH)D and its association with whole-body BMD in a cohort of 16-year-old Icelandic adolescents after adjusting for sex, height, fat and lean mass. We found that 70 % of girls and 66·7 % of boys had 25(OH)D < 50 nmol/l, a higher proportion than previously reported for younger Icelandic children^(6,7)^ but similar to Norwegian adolescents, where 60·2 % had 25(OH)D < 50 nmol/l^(18)^. Adolescents with serum 25(OH)D < 50 nmol/l had significantly lower BMD than those above this threshold value, in agreement with some^(12)^ but not all^(13,14)^ earlier studies on this relationship.

The current findings indicate that the concentration of 25(OH)D in Icelandic children and adolescents decreases with increasing age with 92 % of infants having 25(OH)D above 50 nmol/l^(22)^, 35 % of subjects at the age of 7 and 40 % at the age of 9^(16)^. Similar age-related patterns have been reported among Norwegian children and adolescents^(23)^. At the age of 7 and 9, we did not find an association between bone parameters and 25(OH)D and at those ages the association between PTH and 25(OH)D was weak but significant at age 7 (*r* = –0·21, *P* < 0·05) but insignificant at age 9 (*r* = –0·11, *P* = 0·18)^(16)^. In the current study, the strength of the association has increased to –0·39. This is in agreement with prior studies in adolescents, although it is important to note that the association between 25(OH)D, PTH and bone health in this age group is not fully understood^(24)^.

Our results are in line with some^(12)^ but not all^(13,14)^ previous studies on the relationship between BMD and 25(OH)D in adolescents. For example, a prior Icelandic study did not find an association between 25(OH)D and BMD among 16–20-year-old females^(15)^, although it should be noted that height and lean body mass were not adjusted for as confounding factors, as has been suggested^(25,26)^. In a study among Chinese children and adolescents aged 6–18 years, no association was observed between serum 25(OH)D concentrations and whole-body BMD, but the authors suggest that a dietary enhancement or vitamin D supplementation should be considered for those children who have developed low BMD, to ensure that they can achieve optimal peak bone mass^(27)^.

The observed difference in BMD between those with above *v.* below a 25(OH)D concentration of 50 nmol/l was of about a fifth of one SD, which may have a clinical relevance. In children and adolescents, lower BMD correlates with increased fracture risk during the years of bone accrual, where one sd decrease in volumetric BMD has been associated with a 89 % increase in two years risk of fracture^(28)^. The subjects in the current study are probably in the final stages of a period of peak bone accretion^(29)^, and although the variance of peak bone mass is largely genetically determined, environmental and modifiable factors, such as adequate intake of 25(OH)D, are also important^(30)^ as maximising peak bone mass may contribute to higher BMD later in life and possibly prevent osteoporotic fractures^(31)^.

The main strength of the present study is that all participants came from a single birth year cohort. Many of the participants have previously participated in studies of the association between vitamin D status and bone health^(16)^, allowing us to have insight into the trend of this association within the 1999 cohort. The main limitation of the study is its cross-sectional design. Therefore, no conclusion can be made regarding the causality of the association between the parameters under study. Unfortunately, information regarding nutrition or vitamin D supplementation was not available, as this would have strengthened the study. Furthermore, we only had whole-body BMD but not specific sites that are of particular interest for bone health in adolescents, such as total body less head or the lumbar spine^(32)^. The northern latitude and homogeneous Caucasian composition of the Icelandic population may further limit the generalisability of the findings.

Our results indicate a high prevalence of 25(OH)D insufficiency among Icelandic youth, with lower bone mineral density values observed in those with low 25(OH)D. We conclude that it is important for Icelandic adolescents to increase their vitamin D intake as it is possible that their current intake is insufficient to achieve optimal peak bone mass.
